# Influence of low-temperature nitriding on the strain-induced martensite and laser-quenched austenite in a magnetic encoder made from 304L stainless steel

**DOI:** 10.1038/srep30979

**Published:** 2016-08-05

**Authors:** Vojteh Leskovšek, Matjaž Godec, Peter Kogej

**Affiliations:** 1 Institute of Metals and Technology, Lepi pot 11,1000 Ljubljana, Slovenia; 2 RLS Merilna tehnika d.o.o. Poslovna cona Žeje pri Komendi, Pod vrbami 2, SI-1218 Komenda, Slovenia

## Abstract

We have investigated the possibility of producing a magnetic encoder by an innovative process. Instead of turning grooves in the encoder bar for precise positioning, we incorporated the information in 304L stainless steel by transforming the austenite to martensite after bar extrusion in liquid nitrogen and marking it with a laser, which caused a local transformation of martensite back into austenite. 304L has an excellent corrosion resistance, but a low hardness and poor wear resistance, which limits its range of applications. However, nitriding is a very promising way to enhance the mechanical and magnetic properties. After low-temperature nitriding at 400 °C it is clear that both ε- and α′-martensite are present in the deformed microstructure, indicating the simultaneous stress-induced and strain-induced transformations of the austenite. The effects of a laser surface treatment and the consequent appearance of a non-magnetic phase due to the α′ → γ transformation were investigated. The EDS maps show a high concentration of nitrogen in the alternating hard surface layers of γ_N_ and α′_N_ (expanded austenite and martensite), but no significantly higher concentration of chromium or iron was detected. The high surface hardness of this nitride layer will lead to steels and encoders with better wear and corrosion resistance.

It is well known that austenitic stainless steels cannot be hardened by heat treatments. On the other hand, cold working in combination with a deep cryogenic treatment (DCT) has been shown to be effective at hardening such steels^1^. For example, the hardness of 304L austenitic stainless steel after a combination of cold working and a DCT to form some strain-induced martensite (α′) is above 460 HV_10_. The martensite acts as an elastic reinforcing phase that has a higher stress than the plastically deforming austenite (γ)^1^. Such a material has many potential applications in a variety of areas, including architecture, chemical processing plants and as a magnetic/non-magnetic substrate for encoder scales^2^. The formation of strain-induced martensite is a unique feature of austenitic stainless steels, with two types of martensite being able to form spontaneously in such materials. Early studies^3^,^4^, the results of which were subsequently confirmed in ref. 1, show that the transformation sequence as a result of plastic straining is fcc → hcp → bcc, with the phases being denoted as γ → ε → α′, respectively. The intersections of the stacking faults or the hcp bands act as nuclei for the α′, and if their formation is accelerated, the rate of α′ nucleation is also accelerated. The hcp phase cannot act as an intermediate phase if the composition is changed and the stacking-fault energy is increased; however, such a sequence was found to be applicable in this study. It has also been shown^5^ that the stacking-fault energy in austenitic stainless steel decreases with a decreasing temperature. The formation of strain-induced martensite also occurs at room temperature, but it requires large strains to provide a sufficient thermodynamic driving force. The α′ phase is thermodynamically much more stable than the paramagnetic martensite phase (ε), with the ε phase forming before the α′ phase during the cold working of 304L stainless steel. At high deformations the amount of previously formed ε phase decreases with the increasing deformation because the α′ martensite grows at its expense, i.e., for high levels of deformation the α′ phase dominates the microstructure^6^.

The objective of this research was to develop an innovative process for making a magnetic encoder bar that can be employed for precise positioning using information based on magnetic and non-magnetic phases. Such a bar encoder, which has many applications in the chemical and food-processing industries, made from conventional austenitic 304L stainless steel, has excellent corrosion resistance; however, its low hardness and poor wear resistance limit its range of applications. A low-temperature surface-nitriding process is a possible solution to overcoming this restriction. This means that instead of turning grooves in the encoder bar, which is the usual approach, we transformed the austenite to martensite after a bar extrusion in liquid nitrogen and then marked it with a laser, which caused a very local transformation of the martensite back into austenite. We explored the evolution of the martensite structure in terms of an applied DCT and plastic strain, and revealed how the γ–α′ composite, formed by the deformation, acts as a modifier of the magnetic properties and the mechanical strength, examining the role of the magnetic properties and the hardness of the α′ phase produced by the deformation.

## Results and Discussion

### Vickers hardness HV_10_ and relative magnetic phase (α′) content

The HV_10_ Vickers hardness after vacuum annealing at 1050 °C for 30 min. and then quenching in a flow of nitrogen to room temperature was 160 HV_10_ for the C_−196°C_ bar samples. However, the hardness of the samples in the C_−196°C_ group immersed after each reduction in a bath of liquid nitrogen increased from approximately 295 HV_10_ for the lowest true strain of 0.11 to 395 HV_10_ for the maximum true strain of 0.47. The relative content of martensitic phase (α′) for the C_−196°C_ samples immersed after each reduction in the bath of liquid nitrogen increased in a similar way to the hardness, ranging from approximately 20% α′ for a strain of 0.11 to approximately 60% α′ for a strain of 0.47. This provides clear evidence of a close correlation between a high hardness and a large proportion of α′ phase. As such, a measurement of the hardness after the treatment described above can also provide an indication of the amount of α′ phase in the microstructure of the deformed DCT 304L steel.

A further increase in the amount of martensite α′ in the deformed 304L steel, as reported in ref. 7, can be obtained by annealing at 450 °C, i.e., a few degrees above the austenite transition (A_s_). However, there is no generally agreed explanation for this effect. Mangonon and Thomas^8^ suggested that the increase in α′ at 400 °C was due to the nucleation of new particles, rather than the growth of existing α′ particles, but their explanation is refuted by Guy *et al*.^9^, who attributed the martensite increase at 400 °C to the growth of existing α′ laths, possibly due to the relaxation of the α′ → γ interfaces. It was also suggested^10^ that the increase of α′ in the range 300–400 °C is due to the precipitation of carbides that locally increase M_s_ and so promote additional α′ formation. This suggestion was also refuted by Guy *et al*.^9^, since they did not find any carbide particles. Tavares *et al*.^7^ observed, using isothermal treatments, an increase in the amount of martensite α′ content at 330 °C and 400 °C, and at 550 °C found a small decrease in the martensite content, which suggests an isothermal reversion. At 560 °C a decrease in the magnetization was also observed^7^, indicating that the reaction α′ → γ can progress at a fixed temperature and was not totally athermal. This is in agreement with the observation of Guy *et al*.^9^, that the transformation at 550 °C in an 18Cr–8Ni steel occurred after an incubation period of about 10 min and then progressed by an isothermal diffusion mechanism. The first stage occurred by an athermal shear mechanism.

These findings are very interesting for maximizing the amount of the magnetic phase and the mechanical properties (hardness, etc.) of the deformed DCT 304L steel and avoiding any decrease in the magnetization as well as the hardness due to the reaction α′ → γ, which may progress at approximately 550 °C.

### Metallography and microhardness HV_0.01_

**Figure 1:**
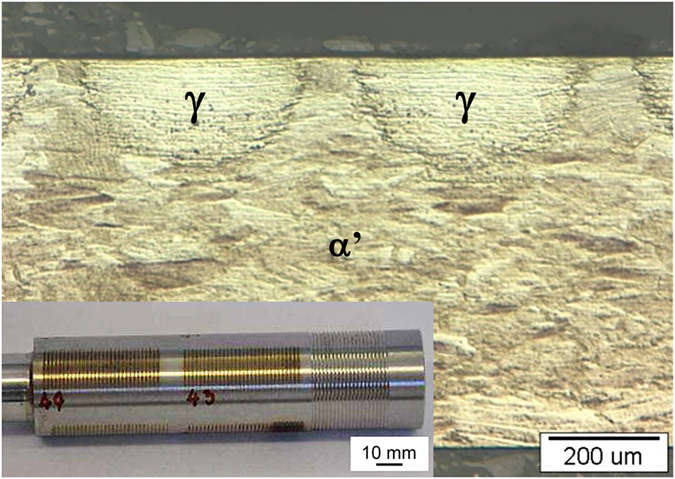
Characteristic profile of the laser marking to a depth of approximately 200 μm and a width of 350 μm in the quenched raster in the shape of a semicircle on the C_−196°C_ substrate for an encoder scale. Inset shows rastered laser surface treatment of rod C_−196°C_.

A metallographic analysis was performed on the C_−196°C_ specimens after cumulative true strains of 0.34 and 0.40 and the laser surface treatment. The microstructure observed before the low-temperature nitriding shows the surface perpendicular to the raster treatment from the laser surface quenching (Fig. 1), with the inset showing the C_−196°C_ rastered rod. The micrograph shows the periodic and uniform semi-circular laser markings of a non-magnetic (γ) plus magnetic (α′) substrate. It is also possible to see the difference in the microstructures between the strain-induced martensite (bulk) and the local non-magnetic phase due to the α′ → γ transformation after the laser surface treatment. This pre-nitriding microstructure clearly shows that the temperature increase on the surface of the rod resulting from the laser was higher than the phase-transformation point, but lower than the melting point.

**Figure 2:**
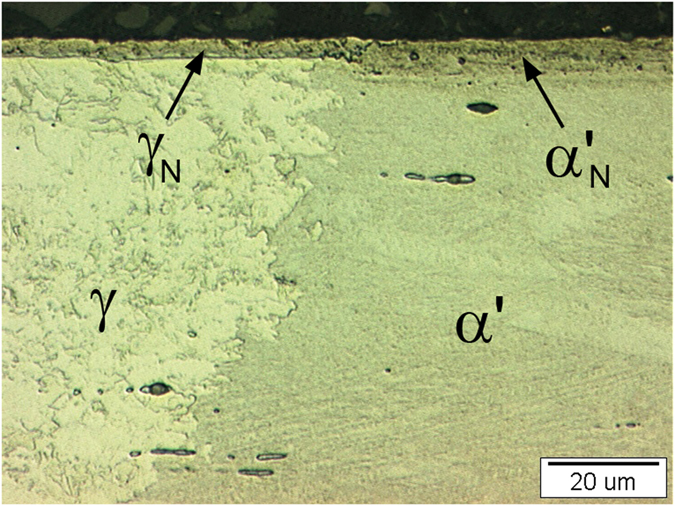
Micrograph of the nitrided layer formed by the pulse plasma nitriding of the AISI 304L strain-induced martensitic (bulk) and the local non-magnetic phase austenitic stainless steel at 400 °C for 10 hours.

Figure 2 is an optical micrograph of a cross-section of the pulse-plasma-nitrided (400 °C for 10 hours), 304L stainless-steel sample with the periodic and uniform semi-circular laser markings of the non-magnetic (γ) plus magnetic (α′) substrate, in which a clear separation can be seen between the martensite and austenite regions in the substrate with a continuous and uniform nitride layer at the top.

The nitrided compound layer is clearly visible, but the thickness of this compound layer for the two different phases is different. For the martensite (α′) the thickness of the compound layer (α′_N_) is 7–8 μm, while the compound layer (γ_N_) above the austenite (γ) it is 3.5–4.0 μm. It is clear that the difference in the thickness of the compound layers is neither a function of the nitriding temperature or time, but depends only on the type of phase (α′ or γ) treated under the same conditions. The different thicknesses are a consequence of the fact that the diffusion coefficient of nitrogen in martensite is much higher than that in austenite^11^, so the martensite layer provides a path for rapid nitrogen transportation in the material. In addition, defect densities (dislocations, grain boundaries) are also important factors that enhance the diffusion of nitrogen in materials. Severe deformation can create much higher defect densities in the strain-induced martensite bulk. Therefore, the nitrogen diffusion depth in 304L for strain-induced martensite is much greater than that of the surface-laser-quenched, strain-induced martensite that transforms into austenite.

Overall, a 3.5–8-μm-thick compound layer was obtained after the nitriding at 400 °C for 10 hours above the austenitic and martensitic phases. The transition of the compound layer from the austenitic microstructure to the martensitic microstructure is smooth and without cracks.

**Figure 3:**
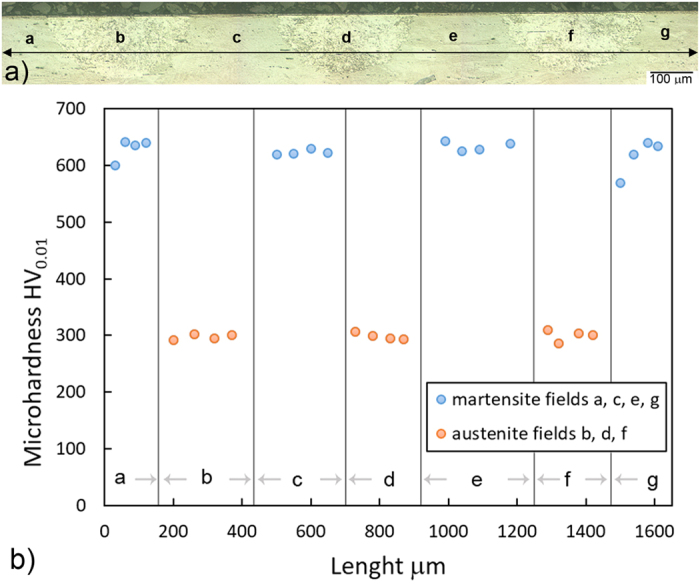
(**a**) Locations of microhardness HV_0.01_ measurements over the martensitic and austenitic fields for the C_−196°C_ specimens at a depth of ~50 μm after low-temperature pulse plasma nitriding in combination with an isothermal treatment, together with (**b**) the variation in the microhardness over the martensitic (a,c,e,g) and austenitic fields (b,d,f) of the C_−196°C_ specimen at a depth of ~50 μm.

Figure 3 shows the range of the microhardness measurements over the martensitic and austenitic phases of the C_−196°C_ specimens at a depth of ~50 μm after the low-temperature pulse-plasma-nitriding in combination with an isothermal treatment for 10 hours. The lower part of the figure shows the values of the microhardness over the martensitic (a, c, e, g) and austenitic phases (b, d, f) of the C_−196°C_ specimen at a depth of ~50 μm, while the upper part has a line showing where the hardness indentations were made.

The average microhardness values of the martensitic phase (α′) and the local non-magnetic phase (γ) formed after the surface laser quenching and the low-temperature pulse plasma nitriding in combination with an isothermal treatment for 10 hours at 400 °C for a depth of ~50 μm are ~620 HV_0.01_ and ~295 HV_0.01_, respectively.

**Figure 4:**
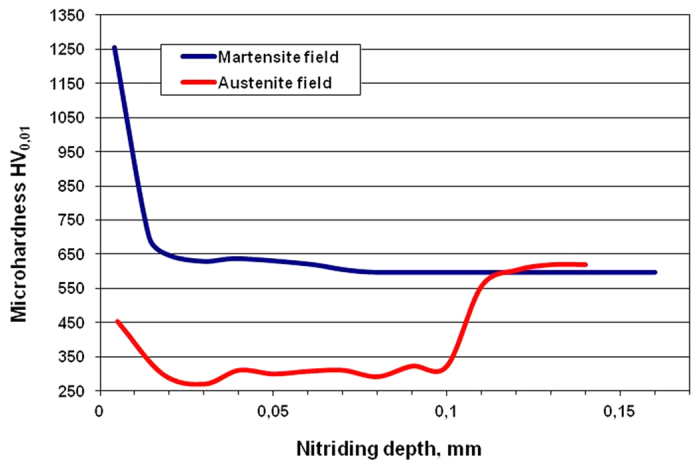
Microhardness depth profile in the martensitic and austenitic fields of the C_−196°C_ specimen.

The laser-surface-treated rods that were plasma nitrided at 400 °C also showed an increase in the hardness by 120 HV_10_ and ~7.5% of relative magnetic phase (α′) content in the bulk microstructure. The transformation sequence as a result of the plastic strain is γ → ε → α′. From this we assume that the increase in the hardness and the percentage of relative magnetic phase (α′) in the bulk microstructure can be attributed to the isothermal transformation during the plasma nitriding of the paramagnetic martensite phase (ε) in the α′ ferromagnetic martensite. Figure 4 shows the variation of the depth-profile microhardness values in the martensitic and austenitic phases of the C_−196°C_ specimen.

The microhardness depth profile of the sample subjected to low-temperature pulse plasma nitriding in combination with the isothermal treatment shows a phase-dependent behaviour. This is because at the nitriding temperature the nitrogen penetrates deeper into the martensitic phase in comparison to the austenitic phase and forms a thicker, modified layer above the martensitic phase. The surface hardness is greatly increased due to the formation of nitrided layers and the increase of the nitrogen concentration in both kinds of microstructure. It is clear that the value of the microhardness increased from about 620 HV_0.01_ for the initial martensitic phase up to about 1250 HV_0.01_, and from ~295 HV_0.01_ up to ~525 HV_0.01_ for the initial austenite phase at a depth of 4 μm below the surface after the plasma nitriding at 400 °C. The surface microhardness is ~1320 HV_0.01_. The hardness measurements indicated that the strain-induced martensite results in an increase in the microhardness of the nitrided layers for the low-temperature nitriding. The effective nitriding depth (Nht) in the martensite field is ~40 μm, while in the austenite field it is between 5 and 18 μm. This confirms that the nitriding layer consists of a compound layer and a diffusion zone. The diffusion zone is at least two times thicker in the strain-induced martensite than in the austenite. At a depth of between 0.1 mm and 0.12 mm the microhardness profile obtained in the austenite field shows a gradual increase in hardness in the transition from an austenite to a martensite microstructure. Such a transition hardness from one microstructure to another is, from the point of view of stress conditions, very favourable, since in bars under load it reduces the potential for crack initiation and rapid crack propagation in a harder and less ductile microstructure such as strain-induced martensite.

The hardness of the nitrided layer is determined by two hardening mechanisms: solid-solution hardening and precipitation hardening. Solid-solution hardening is the most important for austenitic stainless steels, while precipitation hardening is the predominant mechanism for the same steels with a strain-induced martensite^11^. According to the results of S. J. Ji *et al*.^12^, the x-ray diffraction pattern of AISI 304 steel without a severely deformed layer nitrided within the temperature range 380 °C to 420 °C exhibits a series of broad peaks, which shift to lower 2θ values than those for normal austenite, which corresponds to a fcc structure that is similar to the nitrogen-expanded austenite phase previously reported by other researchers^13^,^14^,^15^,^16^,^17^,^18^. The broad peaks labelled γ_N_ (expanded austenite or S phase) at a smaller 2θ angle compared with those of the austenite peaks from the original substrate beneath the modified layer are associated with the nitrogen-rich expanded austenite phase. The peak positions of this nitrogen-rich expanded austenite phase change with the nitrogen concentration in the nitrided layer. This indicates that the major constituent in the nitrided layers has an fcc structure similar to the substrate austenite, but with an expanded lattice. The peak-position shift of the expanded austenite increased with nitriding temperature up to 420 °C. At this temperature, the expanded austenite may be saturated in terms of nitrogen. It is also clear from the spectra that the intensity of the original austenite decreased considerably with the increasing temperature of the nitriding. It is expected that, as the temperature of the processing increases, a thicker nitrided layer is obtained, which in turn will result in an increasing contribution from the γ_N_ phase. Furthermore, the diffraction peaks from the nitrided layers were significantly broader than those obtained from the untreated substrate. Therefore, according to ref. 12, it can be concluded that γ_N_ is the only phase present after plasma nitriding at low temperature.

### SEM and EBSDE of the bulk material

**Figure 5:**
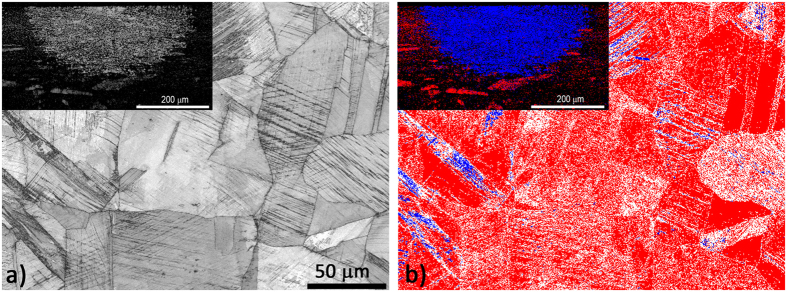
SEM/EBSD analysis of 304L specimen C_−196°C_ sliced perpendicular to the cold-extrusion direction and showing the microstructural changes for a cumulative true strain of ε ~ 0.40–0.46. (**a**) EBSD band-contrast image reveals slip plains forming due to plastic deformation, (**b**) EBSD phase-analysis image (blue for martensite, red for austenite, white for non-indexing patterns). Inset (**a**) shows EBSD image (detail from Fig. 1) of the austenite and martensite phases of the laser-surface-treated C_−196°C_, band-contrast image, while (**b**) shows a phase-analysis image (red for martensite and blue for austenite) ref. 1.

The severity of the cold extrusion at a cumulative true strain of ε ~ 0.40–0.46 could also be observed from the EBSD band-contrast image (Fig. 5a), which clearly indicates that the grains are highly twinned due to the cold extrusion. The EBSD phase analysis (Fig. 5b) showed the formation of a strain-induced martensite phase. Such a phase analysis for a very high deformation rate is not very precise, because the martensite phase is more difficult to index in comparison to the austenite. As a result, most of the non-indexed pixels in Fig. 5b probably belong to the martensite phase.

The phenomenon of a full-hard state exhibited by 304L with a cumulative true strain of ε ~ 0.40–0.46 can be explained in terms of a martensitic transformation by a combination of DCT and cold extrusion. Like with the percentage of martensite formed due to cold extrusion, the hardness of the investigated steel was found to increase after each reduction. When the 304L is cold extruded to a cumulative true strain of ε ~ 0.40–0.46 the volume fraction of martensite is large enough to prevent the formation of any more new martensite by reduction. As a result, the steel is said to be saturated with α′ phase and no additional strengthening is observed, even though a considerable increase in the percentage of prior martensite is seen in the micrograph. However, the relative volume percent of α′ martensite may reach up to ~60 vol. % as a result of a combination of the DCT and the cold extrusion of the 304L. At this deformation level, the parent major austenite phase becomes the minor phase.

According to ref. 19 and based on our microstructural observations above, it can be assumed that dislocation slip, mechanical twinning and martensite transformation co-existed during the deformation of the 304L. It is well known that dislocations are the main carriers of the plastic deformation in crystalline materials, and they may or may not travel across the grains, depending on the nature of the interfaces. However, at all strain levels, dislocation slip is observed to be the dominant deformation mechanism. With increasing plastic strain the dislocation concentration in the grain interiors continues to increase, giving rise to various types of intra-granular dislocation-dislocation interaction processes associated with the glide of extended dislocations on different slip systems. It appears that the dislocation activity is a precursor to the twinning, and subsequently the twinning proceeds as an energetically favourable rearrangement of the partial dislocations (in this case stacking faults).

A series of EBSD measurements was performed to confirm the results obtained from the metallographic examinations. To obtain a representative value for the phase fraction, it is important that the EBSD dataset covers a sufficiently large area and includes enough points. There are two conditions that must be satisfied: the use of the right parameters (number of reflectors, number of bands detected, binning, etc.) for a reliable discrimination of the bcc and fcc phases and, if the microstructure is not homogenous, a large area of analysis. Therefore, an area of 1 mm^2^ was chosen in order to obtain representative data sets. The maps were measured on a grid with a step size of 0.5 μm. Each of the individual beam-scan fields was measured at a magnification of 500×, and due to the very good dynamic focus of the SEM, it was possible to ensure that all the points were in focus. Nevertheless, the problem of a precise discrimination of the martensite and austenite phases becomes difficult, or even impossible, when the amount of highly tetragonal martensite phase increases. The success rate for indexing the austenite is much higher, while the success rate for indexing the tetragonal martensite is lower, and with a high deformation it can be impossible.

As shown in the insets of Fig. 5, i.e., the EBSD analysis of the laser-surface-treated C_−196°C_ after a cumulative true strain of ~0.34 and after a laser surface treatment, the depths and widths of the effects of the heat on the processed volume are approximately 200 μm and 350 μm, and are sufficient for the transformation of the entire strain-induced martensite into austenite, i.e., the light-grey area in Fig. 5. inset a) and the blue area in Fig. 5. inset b).

The microstructure shown in Fig. 1, and confirmed by the EBSD observations in the Fig. 5 insets, shows clearly that each local heat-affected zone is entirely transformed from the strain-induced magnetic martensite phase into the non-magnetic austenite phase. On the surface of the heat-affected zone, no oxide layer, no cast microstructure and no cracks are observed. Thus, it can be concluded that the parameters selected for the laser surface quenching fell within the recommended temperature range of quenching for the 304L austenitic stainless steel.

### SEM and EBSDE of low-temperature plasma nitriding

**Figure 6:**
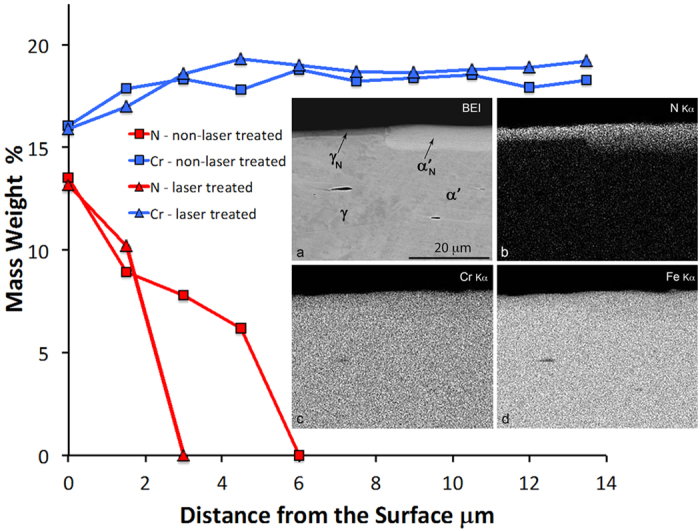
Spot line EDS analyses of Cr and N concentrations from the surface towards the inner region as marked in Fig. 7. Insets show (**a**) backscattered-electron image and corresponding X-ray images of (**b**) N, (**c**) Cr and (**d**) Fe of low-temperature plasma nitriding bar with laser-marked areas.

SEM/EDS/EBSD cross-sections of the low-temperature plasma nitrided bar with laser-marked areas were analysed. The EDS maps of nitrogen, chromium and iron in both surface layers γ_N_ and α′_N_ (expanded austenite and expanded martensite) are shown as insets b), c), d) in Fig. 6. These EDS maps show the high concentration of nitrogen in both surface layers γ_N_ and α′_N_ (expanded austenite and expanded martensite) and no significantly higher concentration of chromium or iron. In fact there is a slight depletion of chromium in both surface layers. The backscattered-electron image shows the different thicknesses of the surface layers formed on the austenite and martensite. There are no carbo-nitride precipitates observed at this magnification in both layers, nor in the martensite and austenite.

The EDS line spot analyses were carried out from the surface to the interior at a distance of 15.5 μm, indicating a greater enrichment of nitrogen in both layers in comparison to the bulk material. The nitrogen distribution follows the different thicknesses of the surface layers, Fig. 6.

Figure 6 shows that the amount of nitrogen is similar in both layers, but drops much more rapidly in the thinner layer. There is no proof of chromium enrichment in either surface layer and, as observed from the mappings, there is a slight depletion of chromium, most probably due to the enrichment of nitrogen. There is a slightly larger amount of chromium in the martensite phase in comparison with the austenite phase.

**Figure 7:**
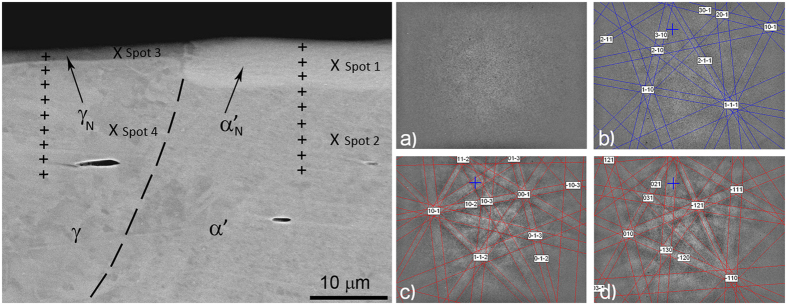
Left: SEM image of the cross-section of the deformed and laser marked DCT 304L steel after low-temperature plasma nitriding, from which a clear separation can be seen between the modified surface region and the substrate (see also Fig. 2). The figure shows the marked spots of the EDS line analyses and the four spots for the EBSD analysis. Right: EBSD Kikuchi patterns reveal that (**a**) the layer formed over the martensite has a very high stress, which is reflected in the highly diffuse pattern–Spot 1, (**b**) strain-induced martensite with a moderately high stress–Spot 2, (**c**) the layer formed over austenite is fcc–identical to the austenite with nitrogen incorporated into the crystal structure–Spot 3, (**d**) laser-treated martensite phase transformed into austenite fcc–Spot 4.

Figure 7 shows a backscattered-electron image on the border between the austenite and martensite phases, with the EDS line spot analyses marked with crosses over both regions of austenite and martensite. Figure 7 also has EBSD spot analyses (X Spot 1, etc.) marked for the four different microstructures. The γ_N_ and α′_N_ layers are clearly visible, but the thickness of the γ_N_ fields is obviously different to that of the α′_N_. The thickness of the α′_N_ is 7–8 μm, while the γ_N_ layer above the area with austenite (γ) is 3.5–4.0 μm. The thinner surface layer of expanded austenite over the austenite phase is darker in the backscattered image than the thicker surface layer of the expanded martensite above the high-stress-induced transformed martensite. The electron-channelling contrast describes the effect of the interaction of energetic electrons with crystalline materials and can be used to detect the crystallographic properties of the sample^20^. The different contrasts of both thin layers can be due to a different chemistry, which was not proven by the EDS analysis, but it can also be due to the channelling effect in relation to the orientation contrast. There is no difference in the chemistry and most probably no significant difference in the preferred crystallographic orientation. The explanation for such a huge difference in the contrast between both layers most probably lies in the different internal stresses caused by the incorporation of nitrogen atoms in the crystal structures. The hardness measurements in Fig. 4 prove this, i.e., the hardness of the expanded martensite is over 1250 HV, while the expanded austenite layer has a hardness of 450 HV. It is known that the fcc structure can incorporate a larger quantity of nitrogen atoms in the interstitial sites, while the bcc structure has fewer interstitial sites. Therefore, the stress in the bcc structure is much higher and this leads to a lighter colour in the backscattered image and more diffuse Kikuchi patterns.

The Kikuchi patterns on the surface-treated area are sharper with less stress, and the thinner surface layer was found to be austenite fcc with an expanded crystal structure (see Fig. 7). The matrix phase is strain-induced martensite, reflected in the moderately highly diffuse Kikuchi patterns. The Kikuchi patterns of the thin surface layer over the martensite that occurred during the low-temperature nitriding are so blurred that no phase could be resolved. High-magnification BEI images show no chromium carbide precipitation.

### From research to innovation

The combination of a deep-cryogenic treatment, plasticity, low-temperature pulse plasma nitriding with an isothermal treatment and phase transitions (ε → α′), in this order, increases the amount of formed magnetic α′ phase, thereby improving the magnetic contrast when applying a non-magnetic γ marking on the substrate by laser surface quenching^1^.

**Figure 8:**
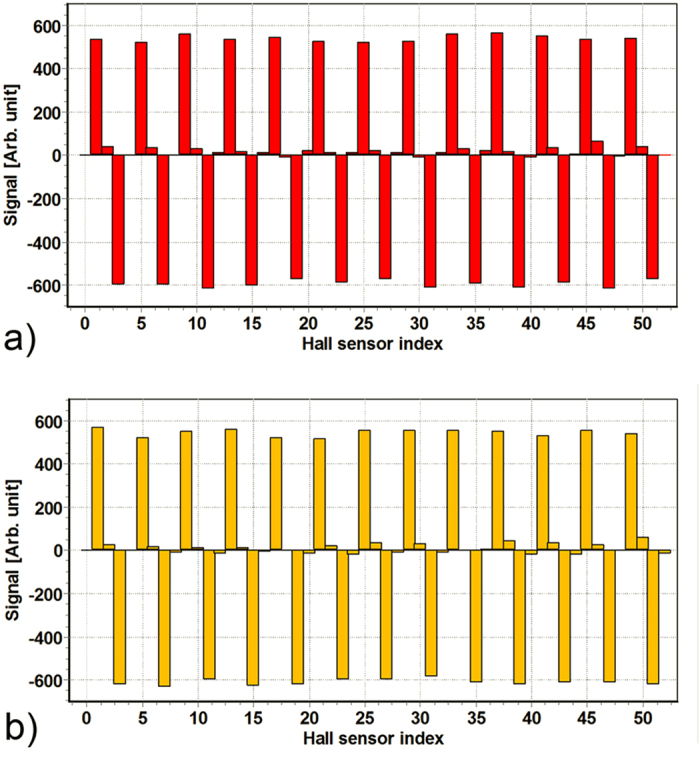
Measurements of the Hall probe on rod C_−196°C_, (**a**) the heat-affected-zones laser surface treatment, (**b**) the grooves made by turning.

Measurements with a Hall probe on the C_−196°C_ rod (Fig. 8a) showed that the amplitude of the signal after the low-temperature pulse plasma nitriding in combination with the isothermal treatment at 400 °C was the same as the amplitude of the signal of a conventional encoder bar with the grooves made by turning and filled by electrolytically deposited, environmentally questionable, hard chromium (Fig. 8b).

From the amplitude of the signal of the Hall probe it can be concluded that the alternating hard surface layers γ_N_ and α′_N_ do not adversely affect the magnetic properties on the substrate for the encoder scale. Furthermore, due to the higher hardness of the austenite as well as the martensite bulk microstructure obtained after the deep-cryogenic treatment and plastic deformation, as well as the relatively thicker layer obtained after low-temperature nitriding in combination with the isothermal treatment, in comparison to the low-temperature nitriding of the same grade of stainless steel with an initial austenite microstructure, it could be expected that the load-bearing capacity of the surface, which is in the latter case limited to mild wear phenomena, will significantly increase.

## Conclusions

The presented results allow us to conclude that the interaction of a deep-cryogenic treatment, plastic deformation, phase transitions, and low-temperature pulse plasma nitriding, in combination with an isothermal treatment as well as laser surface quenching, is a promising area for future work as it allows us to explore new structures and find new applications. In addition, it provides a route to develop materials with an exceptional combination of magnetic properties, strength, ductility, as well as wear and corrosion resistance.

It was shown that an encoder bar, for precise positioning applications, can be produced using an innovative approach. Instead of turning grooves, it is possible to store information in the material by introducing non-magnetic areas with a laser surface treatment. In many applications austenitic stainless steel is used because of its excellent corrosion properties. However, for most encoder-bar applications, a good surface hardness and wear resistance are necessary. Therefore, low-temperature nitriding was proposed. Nitriding causes the formation of expanded martensite over strain-induced martensite with no chromium carbide precipitation. The EBSD Kikuchi patterns show very high stress in the martensite surface layer.

The EDS maps show a high concentration of nitrogen in the alternating hard surface layers of γ_N_ and α′_N_ (expanded austenite and martensite), but no significantly higher concentration of chromium or iron was detected. The high surface hardness of this nitride layer will lead to steels with better wear and corrosion resistance, thus clearly demonstrating the advantages of this superposition of two technologies.

## Experimental Methods

### Material, Cold Working and DCT

The samples used in the investigation were cut from a bar (ϕ18 × 3000 mm) of AISI 304L stainless steel with the composition given in Table 1.

The bar samples, labelled as C_−196°C_, were machined with the axis in the direction of the bar’s extrusion, annealed at 1050 °C for 30 min in a vacuum furnace, and then quenched to room temperature in a flow of high-purity nitrogen to obtain a fully austenitic initial microstructure. The bar extrusion of the C_−196°C_ samples was performed using an experimental drawing bench with eleven exchangeable cold-drawing matrices of different diameters (from 16.72 to 12.19 mm) at liquid-nitrogen temperature. After the immersion of the C_−196°C_ samples with an initial diameter of 18 mm in the liquid nitrogen, nine different diameter reductions were applied, with the partial strains listed in Table 2.

After each reduction, the samples were immersed in liquid nitrogen to equalize the temperature of the drawn bars with that of the liquid nitrogen.

### Laser surface treatment

The experiments were performed on rods (ϕ18 × 80 mm) taken from bar samples of the C_−196°C_ group with the highest content of martensitic phase (α′) using a YLR-400-AC ytterbium-fibre laser machine with a nominal output power of 400 W. The power of the laser in the experiment was set to ~100 W, with the parameters required to obtain an approximate depth of 350 μm and the width of the quenched layer in the shape of a semicircle in the form of a raster, perpendicular to the direction of the bar’s extrusion^11^.

### Low-temperature nitriding

The thermochemical treatment of the plastically deformed, DCT, austenitic, stainless-steel specimens after laser quenching was performed in a Metaplas Ionon HZIW 600/1000 reactor equipped with a convection-heating system and an internal gas/water heat exchanger for rapid cooling. The convection and plasma heating of the specimens to the processing temperature took approximately 3.5 hours. The soaking time at the nitriding temperature of 400 °C, which was also the isothermal treatment temperature, was 10 hours. Before introducing the process gas, the chamber was evacuated to a pressure of 2 Pa. The 4-kHz pulse plasma was produced with a total pressure of 3.3 hPa for nitriding, with a negative bias potential of 490 V being applied to the specimens, which act as the cathode. The specimens were nitrided in a 75 vol-% H_2_: 25 vol-% N_2_ gas mixture.

### Optical and SEM microscopy and Vickers hardness

The optical microscopy, SEM and SEM-based electron-backscatter-diffraction (EBSD) analyses were performed on polished samples to confirm the presence of the magnetic phase (α′) as well as the γ, γ_N_ or α′_N_. The microstructures were examined in an optical microscope (Nikon Microphot FXA) and the EBSD data were collected in a FE-SEM JEOL JSM 6500F field-emission scanning electron microscope using an HKL Nordlys II EBSD camera and Channel 5 software. The EBSD mapping analyses were performed with samples tilted at 70° using a 15-kV accelerating voltage and a 1.5-nA probe current. The EBSD map was generated in steps of 0.4 μm using 613 × 4459 grids with 4 × 4 binning and employing 25 reflectors and minimum 5 and maximum 6 band detection for the best discrimination between the bcc and fcc phases. The analysed area was sufficient to be statistically representative and the selected scanning step was sufficiently small to obtain a clear morphology of the microstructure.

The Vickers hardness HV_10_ and microhardness HV_0.01_ were measured on the same metallographic samples as used for the determination of the relative magnetic phase content (α′) by employing a Vickers Tukon 2100 B hardness tester. The relative volume fractions of the transformed martensite at different stages of the deformation process were assessed using a Ferritescope MP-30.

## Additional Information

**How to cite this article**: Leskovšek, V. *et al*. Influence of low-temperature nitriding on the strain-induced martensite and laser-quenched austenite in a magnetic encoder made from 304L stainless steel. *Sci. Rep.* **6**, 30979; doi: 10.1038/srep30979 (2016).

## Figures and Tables

**Table 1:** Nominal composition (in wt.%) of the alloy used in the investigation.

	C	N	Cr	Ni	Mn	Si	Cu	Mo	Ti
C_−196°C_	0.017	0.08	18.94	8.85	1.32	0.34	0.27	0.23	0.002

**Table 2:** Combination of temperature and plastic strain tests.

Group of samples	Engineering plastic strain applied in a single pass ε_d_ (%)										
	Pass 1	Pass 2	Pass 3	Pass 4	Pass 5	Pass 6	Pass 7	Pass 8	Pass 9	Pass 10	Pass 11
C_−196°C_	13.7	9.7	9.7	10.6	5.3	5.0	2.3	2.8	4.6	5.2	5.7
